# Antimicrobial resistance in plant-associated microbial communities: mechanisms, ecology, and one health implications

**DOI:** 10.3389/fmicb.2026.1778342

**Published:** 2026-08-03

**Authors:** Maddi Sandhya, Eswaran Priyadharshini, Theerthagiri Anand, Nagendran Tharmalingam, Govindasamy Senthilraja

**Affiliations:** 1 Department of Plant Pathology, Centre for Plant Protection Studies, Tamil Nadu Agricultural University, Coimbatore, Tamil Nadu, India; 2 Department of Medicine, Houston Methodist Research Institute, Houston, TX, United States

**Keywords:** antibiotics, antimicrobial resistance, microbiome, one health, phage therapy, RNA based biopesticides

## Abstract

Antimicrobial resistance (AMR) has emerged as a major global challenge threatening human, animal, plant and the environment health. Although AMR has been extensively studied in clinical and veterinary sectors, its emergence and dissemination in plant-based agricultural systems remains comparatively underexplored despite of the widespread use of antibiotics, fungicides and other antimicrobial compounds in crop production. These practices exerts selective pressure on plant associated microbial communities, promoting the evolution and dissemination of antimicrobial resistance genes (ARGs) across interconnected environmental compartments including soil, water, plants and food systems highlighting the importance of one health. This review synthesizes current knowledge on the occurrence, mechanisms and ecological drivers of AMR in plant associated microbial communities. It discusses resistance mechanism in bacterial and fungal plant pathogens, the role of soil, rhizosphere, and phyllosphere microbiomes as reservoirs and transmission hubs for ARGs and evidence from global and regional studies illustrating the complexity of AMR in agricultural ecosystems. The review also evaluates emerging sustainable alternatives to conventional antimicrobial use including phage therapy, microbiome engineering, RNA-based biopesticides, CRISPR-based technologies, and nanotechnology based approaches, which have the potential to reduce chemical reliance and mitigate selection pressure for resistance. Overall, integrating plant health into one health governance frameworks, strengthening AMR surveillance in agricultural systems, and promoting sustainable disease management strategies are essential for limiting the spread of resistance and safeguarding agricultural productivity, ecosystem integrity and public health.

## 1 Introduction

AMR is defined as the ability of microorganisms to survive or proliferate against exposure to antimicrobial agents that were previously effective, has emerged as one of the most significant global challenges affecting human, animal and environment health, with broad implications for food security and sustainable development (Wellington et al., 2013; Larsson and Flach, 2022; Pattis et al., 2022; Shafiq et al., 2025; WHO, 2024; Shafiq et al., 2025). The increasing prevalence and persistence of AMR organisms compromise disease control strategies, reduce treatment efficacy, and intensify economic and public burdens worldwide (McEwen and Collignon, 2018; Velazquez-Meza et al., 2022; Jin et al., 2022; Nashwan et al., 2024; WHO, 2023). Consequently, international agencies have emphasized the need for integrated, cross-sectoral approaches to mitigate the emergence and spread of AMR.

The one health framework has gained prominence as an integrative approach for addressing AMR by recognizing the dynamic interactions among human, animal, and environmental systems (McEwen and Collignon, 2018; Velazquez-Meza et al., 2022; WHO, 2024). Within this framework, AMR is no longer viewed solely as a clinical concern but as an ecological process shaped by antimicrobial use, environmental transmission pathways, and the movement of resistance determinants across interconnected ecosystems (Miller et al., 2022; Jin et al., 2022; Franklin et al., 2024). By emphasizing the movement and persistence of antimicrobials and resistance genes within agro-ecosystems and beyond (Singer et al., 2016; Larsson and Flach, 2022; Shafiq et al., 2025; Geneva Environment Network, 2023), this framework promotes interdisciplinary, policy-coordinated, and stewardship-driven responses to AMR (Nashwan et al., 2024; Velazquez-Meza et al., 2022).

Although agriculture has historically received less attention than clinical and livestock sectors in AMR discussions, increasing evidence identifies crop production systems and plant health management practices as important contributors to resistance emergence and its further spread (McManus et al., 2002; Miller et al., 2022; Verhaegen et al., 2023; Batuman et al., 2024). The application of antibiotics, fungicides, and other antimicrobial inputs in plant disease management imposes selective pressure on plant-associated microbial communities and may alter the composition and function of soil, rhizosphere, phyllosphere, and aquatic microbiomes (Taylor and Reeder, 2020; Shafiq et al., 2025; Kalpana et al., 2024). These environments can act as reservoirs of ARGs, facilitating their persistence and transfer through various mechanisms (Wellington et al., 2013; Singer et al., 2016; Larsson and Flach, 2022; Shafiq et al., 2025).

Plant pathology represents an important but often underrepresented component of AMR management because crop disease control practices directly influence microbial selection dynamics across agricultural ecosystems (Miller et al., 2022; Islam et al., 2024; Kalpana et al., 2024; Franklin et al., 2024). The application of antibiotics and other antimicrobial inputs for phytopathogen management can alter microbial community composition in soil and water habitat, increase selective pressure, and promote the enrichment and persistence of ARGs within plant-associated microbiomes (McManus et al., 2002; Verhaegen et al., 2023; Shafiq et al., 2025). These environments may facilitate the persistence and movement of resistance determinants across environmental matrices through runoff, irrigation networks, and microbial transfer. Therefore, integrating plant pathology into AMR surveillance and stewardship frameworks is essential for developing sustainable disease management approaches that support crop productivity while reducing ecological and public health risks (Miller et al., 2022; Kalpana et al., 2023, 2024; McEwen and Collignon, 2018; Food and Agriculture Organization of the United Nations, 2021; Geneva Environment Network, 2023).

Plant-associated microbial ecosystems therefore represent a critical yet underexplored interface in the ecology of AMR. Unlike clinical environments, resistance dynamics in plant systems are governed by complex ecological interactions involving host plants, microbial communities, environmental conditions, and agricultural interventions. Despite growing recognition of these processes, current understanding remains fragmented regarding how plant-associated resistomes develop, persist, and contribute to broader one health transmission networks. Accordingly, this review critically examines the mechanisms driving AMR within plant-associated microbial communities, evaluates their ecological, one health implications, and discusses emerging strategies for surveillance and mitigation while considering their practical limitations and implementation challenges.

This study was conducted as a narrative review to summarize current knowledge on AMR in plant-associated microbiomes within the one health framework. A structured literature search was performed using Web of Science, Scopus, PubMed, and Google Scholar to identify relevant peer-reviewed articles published between 2000 and 2025. The search employed keywords including “antimicrobial resistance,” “plant microbiome,” “antibiotic resistance genes,” “plant-associated microorganisms,” “plant disease management,” and “one health.” Studies published in English that focused on AMR in plant-associated microorganisms, agricultural ecosystems, resistance mechanisms, or sustainable disease management strategies were included. Articles unrelated to plant or agricultural systems, non-peer-reviewed publications, conference abstracts, and editorials were excluded. The retrieved studies were screened based on their titles, abstracts, and full texts for relevance to the review objectives.

## 2 Sources and drivers of AMR in plant associated microbial communities

### 2.1 Antimicrobial compounds and selective pressure in agricultural ecosystems

Antimicrobial inputs, particularly antibiotics and bactericidal compounds have long been incorporated into crop protection strategies to manage economically important bacterial diseases. Since their introduction into agriculture during the mid-20th century these compounds have contributed to disease suppression and crop productivity. However, their continued and repeated use has also generated concerns regarding AMR development within agricultural ecosystems (McManus et al., 2002; Sundin and Wang, 2018; Miller et al., 2022). Unlike clinical environments where antimicrobial exposure is comparatively controlled, agricultural applications occur under open environmental conditions that facilitate the persistence and spread of AMR.

The use of antibiotics in plant disease management was initiated with streptomycin for the control of fire blight caused by *Erwinia amylovora* in apple and pear production systems (Emeriewen et al., 2019; McManus et al., 2002). Subsequently, additional compounds including oxytetracycline and kasugamycin were introduced for managing bacterial diseases across fruit and horticultural crops (Sundin and Wang, 2018; Stockwell and Duffy, 2012).

Similar practices have expanded to citrus production for managing huanglongbing associated with *Candidatus Liberibacter asiaticus*, and to rice cultivation where antibiotic mixtures such as streptocycline have been used against bacterial blight and leaf streak caused by *Xanthomonas* spp. (Blaustein et al., 2018; Zhang et al., 2014; Qi et al., 2025; Sundin and Wang, 2018). Antibiotics are also used to manage bacterial diseases including leaf spots, cankers, wilts in vegetables and ornamental crops are occasionally incorporated into Integrated Pest Management (IPM) programs for high-value cropping systems experiencing substantial bacterial disease pressure (Stockwell and Duffy, 2012; Miller et al., 2022).

The antimicrobials are commonly applied through foliar sprays, trunk injections and root drenches to provide preventive or curative control, primarily targeting gram-negative and selected gram-positive phytopathogenic bacteria (McManus et al., 2002; Sundin and Wang, 2018). However, repeated incorporation of antibiotics within IPM systems may increase exposure to antimicrobial compounds in plant-associated microbial communities, generating evolutionary pressure that promotes resistant populations and contributes to the persistence and enrichment of ARGs in agricultural environments.

### 2.2 Fungicides

**Figure 1:**
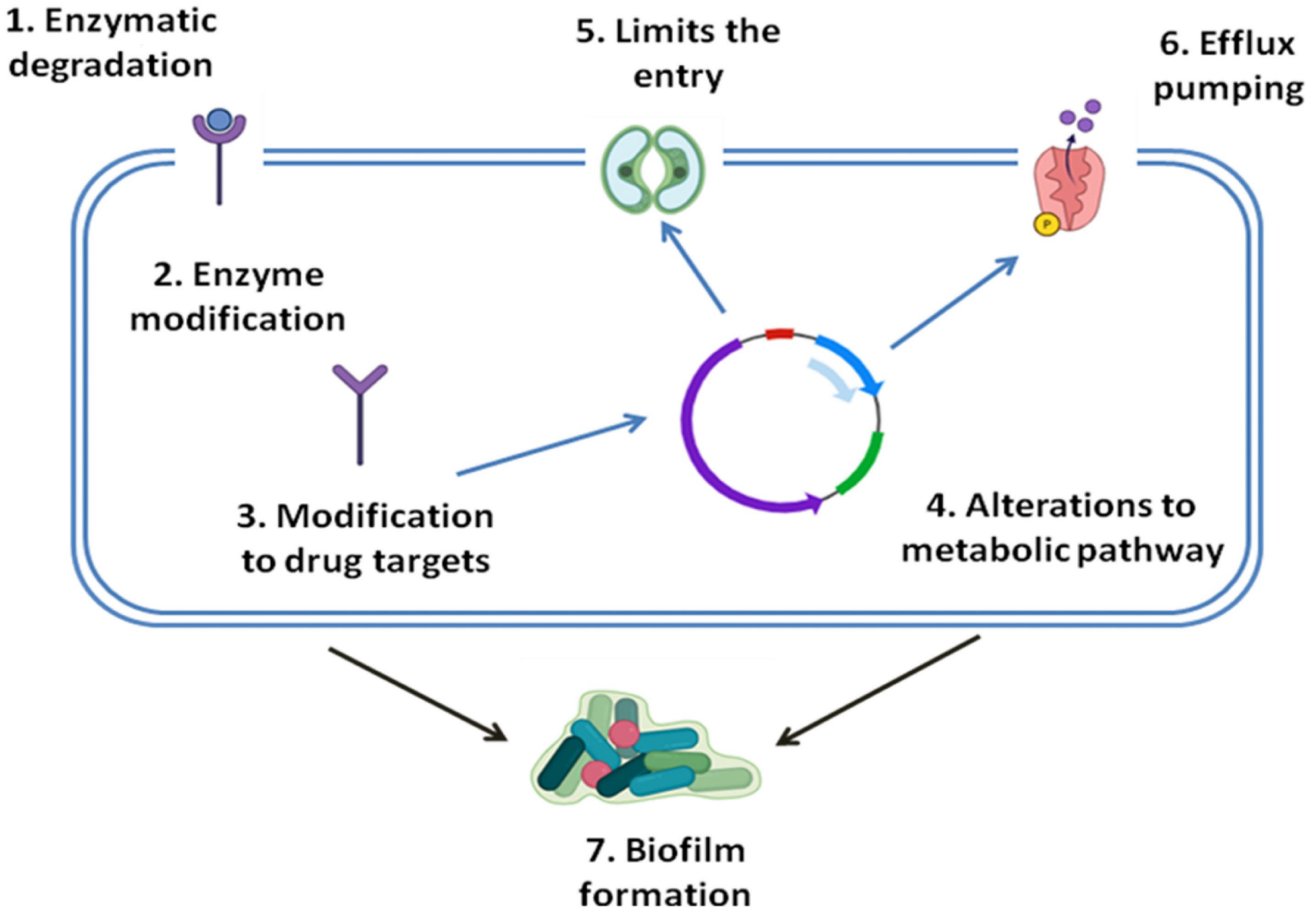
Major mechanisms contributing to antimicrobial resistance in plant-associated microbial communities. The schematic illustrates the principal mechanisms by which bacteria develop antimicrobial resistance, including enzymatic degradation, enzyme modification, and modification of drug targets, alterations to metabolic pathways, limiting drug entry, active efflux pumping, and biofilm formation.

Fungicides remain an essential component of crop protection programs and are extensively applied to manage fungal diseases that reduce agricultural productivity. Among the commonly used classes, benzimidazole fungicides such as carbendazim provide systemic activity against pathogens including *Fusarium*, *Alternaria*, and *Rhizoctonia* species (Ma and Michailides, 2005). Triazole fungicides, including tebuconazole and propiconazole act by inhibiting sterol 14α-demethylase and disrupts ergosterol biosynthesis, making them effective for controlling diseases such as powdery mildew and rust (Price et al., 2015; Cools and Fraaije, 2013). Although fungicides differ mechanistically and chemically from antibiotics, growing evidence suggests that intensive and repeated fungicide application may contribute to AMR development through indirect ecological effects and selection processes within plant-associated microbial communities. Fungicide exposure can alter microbial diversity, modify community composition, and create selective environments that favor resistant microorganisms and resistance-associated traits (Van Bruggen et al., 2019; Sundin and Wang, 2018). In addition, co-selection and cross-resistance mechanisms including activation of multidrug efflux systems, target-site alterations, and adaptive stress responses may contribute to broader resistance development across agricultural ecosystems. This is illustrated in Figure 1. The consequences of prolonged antimicrobial use have been observed across several agriculturally important phytopathogens, including *Xanthomonas*, *Pseudomonas*, *Ralstonia*, *Erwinia*, and *Fusarium*, where reduced sensitivity and resistance have increasingly been reported (Sundin and Wang, 2018; Cools and Fraaije, 2013; Ma and Michailides, 2005). These observations highlight that resistance emergence in crop production systems should be considered not only as a treatment failure issue but also as an ecological process influenced by sustained chemical selection pressure.

In India, antimicrobial inputs continue to contribute to the management of bacterial diseases in staple and horticultural crops. Streptocycline, a combination of streptomycin and tetracycline, has been employed against diseases such as bacterial blight and bacterial wilt in certain agricultural contexts (Miller et al., 2022; Sundin and Wang, 2018). Nevertheless, sustained use of such antimicrobials highlights the importance of stewardship practices that maintain disease control while reducing selection for AMR.

### 2.3 Copper compounds and co-selection

Copper-based fungicides and bactericides are commonly used for routine disease management in vegetable and fruit farming systems for the management of bacterial and fungal diseases (Lamichhane et al., 2018). However, prolonged and intensive application of copper-based compounds has been linked to the emergence of copper-resistant phytopathogens, including *Xanthomonas*, *Pseudomonas,*and *Erwinia*, while also promoting co-selection processes that may enhance the persistence and spread of resistance traits in environmental microbial communities. Such trends raise concerns about the contribution of copper use to broader AMR dynamics within agroecosystems (Lamichhane et al., 2018; Sundin and Wang, 2018; Fan et al., 2022). Environmental monitoring studies have shown that residues of agricultural antimicrobials, particularly streptomycin and oxytetracycline, can persist in soils and irrigation water beyond their original application areas (Wu et al., 2023; Ren et al., 2023). These residues can act as selective agents within plant-associated and environmental microbiomes, promoting shifts in microbial community structure and increasing opportunities for resistance enrichment (Singer et al., 2016).

Co-selection represents an additional challenge, as resistance to metals and antimicrobials can become genetically linked. Copper resistance determinants and ARGs are frequently associated on shared mobile genetic elements, facilitating their co-maintenance and dissemination under copper selection pressures even in the absence of antibiotic use (Seiler and Berendonk, 2012; Baker-Austin et al., 2006).

### 2.4 Cross resistance from non-antibiotic chemicals

Increasing evidence suggests that AMR in agricultural environments is not driven exclusively by antibiotic exposure but also by non-antibiotic chemical inputs that impose overlapping or indirect selection pressures. Compounds including copper, zinc, quaternary ammonium compounds, glyphosate and azole based fungicides have been implicated in promoting resistance development through mechanisms of cross-resistance and co-selection (Seiler and Berendonk, 2012; Baker-Austin et al., 2006; Liao et al., 2021).

Cross-resistance occurs when a single resistance mechanism such as multidrug efflux systems or changes in membrane permeability confers reduced susceptibility to multiple classes of compounds, whereas co-selection occurs when resistance determinants for different stressors are genetically linked and maintained together. In plant-associated and environmental microbiomes, resistance genes associated with metals and other environmental co-selective agents, such as biocides and disinfectants, are frequently co-localized with ARGs on mobile genetic elements, including plasmids and transposons (Seiler and Berendonk, 2012; Baker-Austin et al., 2006). As a result, exposure to non-antibiotic compounds may sustain and enrich resistance even in the absence of direct antibiotic application.

Studies investigating agricultural ecosystems have reported widespread occurrence and enrichment of ARGs following exposure to multiple chemical classes, indicating that resistance selection extends beyond conventional antimicrobial use (Kalpana et al., 2024; Wu et al., 2023; Ren et al., 2023). These chemical-induced shifts may alter microbial community composition and increase opportunities for horizontal gene transfer (HGT) among crop-associated microorganisms and surrounding soil and water microbiota (Singer et al., 2016; Baker-Austin et al., 2006). Consequently, resistance emergence in agricultural systems should be considered an ecosystem-level process rather than a direct consequence of antibiotic application alone.

Despite increasing recognition of these interactions, regulatory oversight of antimicrobial and chemically mediated resistance selection in crop production remains limited in many regions. Strengthening resistance management frameworks through evidence-based compound approval, optimized application timing, dosage management, and integration with resistance surveillance programs may reduce unnecessary selection pressure while supporting sustainable disease management practices (Miller et al., 2022; Van Bruggen et al., 2019).

## 3 Mechanisms of AMR development and dissemination

AMR in plant pathogens is a multifaceted issue influenced by a variety of molecular, genetic, and ecological factors. These functions enable pathogens to survive antimicrobial treatment, leading to prolonged plant diseases and the widespread of resistance genes within natural and agricultural environments. This synthesis integrates knowledge on interconnected mechanisms driving AMR in plant associated environments, including point mutations, plasmid-mediated gene transfer, efflux pump activity, enzymatic inactivation of antimicrobials, and metabolic defense systems. It also highlights how ecological selection imposed by antimicrobial use, together with community-level adaptations such as biofilm formation and quorum sensing, enhances microbial persistence, facilitates the exchange of resistance genes, and accelerates the emergence and dissemination of AMR within plant microbiomes (Table 1) These processes are discussed within the plant health dimension of the one health concept (Sundin and Wang, 2018; Van Bruggen et al., 2019).

**Table 1:** Antimicrobial compounds, resistant plant pathogens, and the associated resistance mechanisms.

S. no	Antimicrobial chemical	Type	Plant pathogens exhibiting resistance	Mechanism of resistance	Reference
1.	Streptomycin	Antibiotic	*Erwinia amylovora*, *Pseudomonas syringae*, *Xanthomonas campestris*, *Clavibacter michiganensis*	*rpsL* mutations, *strA/strB* acquisition via plasmids	McEwen and Collignon (2018) and Sundin and Wang (2018)
2.	Oxytetracycline	Antibiotic	*Candidatus* Liberibacter asiaticus, *Xanthomonas* spp.	Efflux pumps, ribosomal protection genes	Zhang et al. (2014)
3.	Copper compounds (CuSO₄, Cu(OH)₂, CuO)	Bactericide/Fungicide	*Xanthomonas* spp., *Pseudomonas syringae*, *Erwinia amylovora*, *Alternaria alternata*, *Colletotrichum* spp.	Copper tolerance via *copLAB*, *cueO*, biofilm formation	Lamichhane et al. (2018) and Behlau et al. (2013)
4.	Azoles (DMIs) e.g., tebuconazole, propiconazole	Fungicide	*Fusarium* spp., *Alternaria alternata*, *Aspergillus fumigatus*, *Cercospora beticola*	*CYP51* mutations, overexpression, efflux pumps	Snelders et al. (2008) and Ghannoum (2016)
5.	QoI fungicides (strobilurins) e.g., azoxystrobin, pyraclostrobin	Fungicide	*Botrytis cinerea*, *Alternaria solani*, *Plasmopara viticola*, *Fusarium graminearum*	G143A / F129L mutations in *cytb* gene	Shcherbakova (2019)
6.	SDHI fungicides, e.g., fluxapyroxad, boscalid	Fungicide	*Botrytis cinerea*, *Alternaria alternata*, *Zymoseptoria tritici*, *Fusarium asiaticum*	Mutations in *sdhB*, *sdhC*, *sdhD*	Avenot and Michailides (2010)
7.	Benzimidazoles (carbendazim, benomyl)	Fungicide	*Colletotrichum gloeosporioides*, *Botrytis cinerea*, *Rhizoctonia solani*, *Fusarium oxysporum*	β-tubulin mutations (E198A/K, F200Y)	Quaranta et al. (2012) and Ma and Michailides (2005)
8.	Phenylamides (metalaxyl, mefenoxam)	Fungicide	*Phytophthora infestans*, *P. capsici*, *Pythium ultimum*	Point mutations in *Avr3a* and RNA polymerase genes	Gisi and Sierotzki (2015)
9.	Dithiocarbamates (mancozeb)	Fungicide	*Alternaria alternata*, *Cercospora beticola*	Detoxification enzymes, efflux mechanisms	Thind and Hollomon (2018)
10.	Quaternary ammonium compounds (QACs)	Disinfectant/Biocide	*Xanthomonas* spp., *Pseudomonas syringae*, *Agrobacterium tumefaciens*	Membrane adaptation, efflux pumps	Hegstad et al. (2010)
11.	Phosphites (phosphonates)	Fungicide	*Phytophthora infestans*, *P. cinnamomi*	Modified phosphate uptake pathways	Gent et al. (2020)
12.	Sulfur fungicides	Fungicide	*Oidium/Erysiphe* spp. (reduced sensitivity observed)	Altered membrane permeability, detoxification	Baibakova et al. (2019)

### 3.1 Genetic mechanism

#### 3.1.1 Mutation-based resistance

The acquisition of phyto-pathogenic resistance is often initiated by mutations in chromosomal loci that encode antibiotic targets. Streptomycin, one of the earliest antibiotics used in the control of plant diseases, inhibits protein synthesis by binding to the 30S ribosomal subunit and interacting with 16S rRNA and ribosomal protein S12 (Springer et al., 2001). Resistance results from mutations in rpsL (which encodes ribosomal protein S12) or rrs (which encodes 16S rRNA), which reduce antibiotic binding without primarily compromising ribosomal function. Point mutations of this kind have been identified in the causal agents *E. amylovora, X. campestris* and *P. syringae*, resulting in large populations of streptomycin-resistant strains in agricultural fields (Sundin and Wang, 2018; Chiou and Jones, 1995; Jones and Schnabel, 2000).

Tetracyclines exert their effects by preventing aminoacyl-tRNA from binding to the A-site of the ribosome once they are bound to the 30S subunit, without affecting the peptidyltransferase binding site on the 50S subunit. Mechanisms of resistance may result from mutations that decrease drug affinity, or from the acquisition of ribosomal protection proteins (RPPs), such as those encoded by *Tet* genes. These proteins displace tetracycline molecules from ribosomes, thereby re-initiating translation (Roberts, 2005). Such genetic changes may arise spontaneously in the presence of selective antibiotic pressure and become established in populations that are in permanent contact with these chemicals.

#### 3.1.2 Plasmids and HGT mechanisms

In addition to chromosomal mutations, the active horizontal transfer of genes promotes the evolution of resistance in phytopathogens. Plasmids are autonomous extrachromosomal DNA molecules that often harbor multiple resistance genes, and they may be capable of transferring these genes by conjugation, thereby accelerating the spread of resistance within and between species (McManus et al., 2002; Sundin and Wang, 2018). This type of plasmid-borne resistance enables rapid adaptation to antimicrobial therapy and the co-localization of genes encoding various resistances. A wide range of bacterial genera, including *Xanthomonas*, *Pseudomonas*, *Ralstonia*, *Agrobacterium*, *Enterobacter*, *Klebsiella* and *Escherichia* have been reported to harbor self-transmissible plasmids that facilitate the horizontal transfer and dissemination of ARGs within and across bacterial populations (Sundin and Wang, 2018).

Resistance gene cassettes are transferred to plasmids or chromosomes through HGT mechanisms mediated by mobile genetic elements such as integrons and transposons. Bacteriophages facilitate the dissemination of drug resistance via transduction, involving the transfer of genetic material between bacteria (Balogh et al., 2010; Javeedvali et al., 2024). These gene exchange pathways enable the widespread distribution of AMR genes among plant pathogens, as well as across soil, water and microbes associated with humans. This forms a genetic link between environmental reservoirs and clinical AMR cases. These observations highlight significant one health intersections due to the mobility and persistence of these genes within agricultural ecosystems (Singer et al., 2016; Van Bruggen et al., 2019).

### 3.2 Biochemical resistance mechanisms

Phytopathogens employ several biochemical strategies to resist antimicrobials. One such strategy is the use of efflux pumps, which belong to different gene families, such as the Resistance-Nodulation-Division (RND) family of transporters. These pumps actively remove antibiotics from bacterial cells, thereby lowering intracellular concentrations to sub-lethal levels (Nikaido, 2018). These pumps often exhibit non-susceptibility to multiple antibiotic classes simultaneously, which complicates efforts to control drug resistance. Another resistance mechanism involves enzymatic denaturation. The streptomycin-inactivating enzyme, for example, is an aminoglycoside phosphotransferase that chemically alters antibiotics, reducing their ability to bind to ribosomes (Chiou and Jones, 1995). Tetracycline-inactivating enzymes and ribosomal protection proteins (RPPs) also effectively protect the translational machinery (Roberts, 2005). These enzymatic processes are often encoded by plasmids, enabling easy transfer to other bacteria in agricultural environments.

### 3.3 Community interactions: biofilms and quorum sensing

In addition to individual resistance strategies, microbial population can demonstrate collective resistance by forming biofilms and engaging in quorum sensing. Biofilms are dense aggregations of bacteria surrounded by extracellular polymeric substances (EPS), which coat surfaces such as plant leaves, soil particles and irrigation systems. The bacterial cells within these biofilms undergo physiological changes, including decreased metabolic activity and increased stress, which makes them less vulnerable to antibiotics.  The EPS matrix acts as a diffusion barrier, restricting the entry of antimicrobial agents and creating a microenvironment in which bacteria can survive even at high antibiotic concentrations. The close proximity of cells in biofilms promotes the exchange of resistance genes through HGT (Ramey et al., 2004). Quorum sensing (QS) is a bacterial communication system in which signaling molecules, such as N-acyl homoserine lactones (AHLs), are used by Gram-negative bacteria to control biofilm formation, as well as the activation of resistance and virulence genes. QS co-ordinates group actions, enabling pathogen populations to launch a unified defense against antimicrobial agents. Disrupting QS has been considered a promising approach to combat biofilm formation and antibiotic resistance (Chagas et al., 2018).

### 3.4 Physiological and ecological mechanisms

Resistance selection and evolution occur in environments where livestock production takes place and there is constant antimicrobial exposure. Antibiotics and fungicides are often applied intensively, with or without regulation, to control crop diseases, leading to the fixation of resistance mutations and the transmission of resistance plasmids. The continued detection of antibiotic residues in soils and irrigation water indicates ongoing environmental exposure, which can serve as a migratory source of resistance genes between microbial communities (Wu et al., 2023; Ren et al., 2023; Lamichhane et al., 2018). This diverse resistance terrain, involving pathogen adaptation, the environment, and human activity, requires coordinated surveillance and stewardship strategies in line with the one health approach. Combating AMR dissemination, which threatens agro-ecosystem, human, and animal health, requires an integrated approach involving plant pathology, microbiology, environmental science and public health, along with the implementation of comprehensive plant protection measures (Van Bruggen et al., 2019).

### 3.5 Plant microbiome as a reservoir and conduit of AMR

Agro ecosystems represent interconnected reservoirs and transmission networks for remnants of antibiotics, resistant microorganisms, and resistance genes. In this section, we develop the environmental paths including soil, water, air and crop surfaces and relate them to plant, animal and human health, while recognizing the complexity of transmission of AMR under a one health perspective (Singer et al., 2016; Van Bruggen et al., 2019).

#### 3.5.1 Soil as an AMR reservoir

Soils, particularly those in agricultural settings, can serve as important reservoirs of both antibiotics and antibiotic-resistant microorganisms. When antibiotics and fungicides are used for crop protection, their residues accumulate in the soil matrix, consequently interacting with the indigenous microbiome. Studies have shown that antibiotic residues can significantly impact the composition and activity of soil microbiota at sub-inhibitory levels, increasing the abundance of resistant taxa while reducing sensitive ones (Ren et al., 2023; Wu et al., 2023). These changes affect the ecological equilibrium, impacting plant nutrition, health and microbial diversity (Udikovic-Kolic et al., 2014).

Antibiotic residues and antibiotic resistance genes can persist in soil microbiomes for a long time after the cessation of antibiotic use; this phenomenon is known as the ‘legacy effect’. Past exposure continues to shape the soil resistome (Udikovic-Kolic et al., 2014). AMR associated genes in soil rely on mobile genetic elements, which makes them transmissible between bacteria, including both plant- and human-associated pathogens (Zhu et al., 2013; Van Bruggen et al., 2019). This makes soil a major AMR hotspot, with an impact on agriculture, human health, and animal health.

#### 3.5.2 Rhizosphere and phyllosphere communities

The rhizosphere is a hotspot for microbial diversity and activity, supporting intense microbial interactions and mobile gene transfer. Root exudates shape rhizosphere microbial community assembly by providing chemical cues and nutrient substrates that influence microbial proliferation and interactions (Fitzpatrick et al., 2020). The presence of antimicrobial compounds and their residues in soil and irrigation water can impose selective pressure on microbial communities, therby facilitating the persistence and dissemination of ARGs within soil–plant systems through HGT mechanisms (Lu et al., 2021; Ren et al., 2023). Wu et al. (2024) further highlighted that soil, irrigation water, fertilizers and plant associated microbiota serve as important reservoirs and transmission pathways for ARGs in agricultural ecosystem. In the phyllosphere, antimicrobial residues deposited through foliar applications, airborne deposition and water splashes may enrich ARG- harbouring microorganisms and contribute to the spread of resistant bacteria within the plant environment (Thapa and Prasanna, 2018).

#### 3.5.3 Water mediated transmission

The agricultural fields and the surrounding environment are interconnected through water systems, which can facilitate the evironemnetal dissemination of AMR across different ecosystems. Surface runoff and irrigation water may transport ARGs and antibiotic-resistant bacteria from agricultural fields to streams, rivers, ponds, and other water bodies (Pruden et al., 2012; Berendonk et al., 2015). Fito and Van Hulle (2021) reported that irrigation water containing antibiotic residues or ARGs may contribute to the introduction of resistant bacteria into crop associated microbial communities. These findings highlight water as a potential pathway for the environmental spread of AMR rather than confirming direct transmission to humans. Therefore, a one health approach is essential to understand and monitor the interconnected movement of AMR determinants across agricultural and environmental compartments (Van Bruggen et al., 2019).

## 4 One health perspectives and environmental linkages

Plant-associated environments are increasingly recognized as important interfaces connecting environmental, animal, and human AMR reservoirs. Rather than functioning as isolated compartments, agricultural ecosystems contribute to a connected resistome shaped by microbial exchange, environmental transport, and anthropogenic activities (Berendonk et al., 2015; Singer et al., 2016). Advances in metagenomics and molecular epidemiology studies have revealed shared resistance genes and mobile genetic elements across bacteria isolated from plants, soils, animals, and human-associated environments (Zhu et al., 2013; Negreanu et al., 2012; Van Bruggen et al., 2019). Mobile elements carrying resistance determinants, including *β*-lactamases and aminoglycoside-modifying enzymes, have been detected across multiple ecological compartments, indicating opportunities for HGT and long-term resistance maintenance. Similar resistance profiles have also been reported in bacterial genera such as *Escherichia coli*, *Enterobacter*, and *Pseudomonas* recovered from both agricultural and clinical contexts, suggesting that agricultural environments may contribute to broader resistance circulation networks (Yang et al., 2024; Badawy et al., 2023). Agricultural practices can further strengthen these connections. For example, the use of wastewater for irrigation may introduce resistant microorganisms, antimicrobial residues, and ARGs into crop production systems, increasing opportunities for microbial exchange among soil, plants, water, animals, and human populations (Fito and Van Hulle, 2021; Pruden et al., 2012).  Understanding the pathways through which resistance emerges, persists, and disseminates across plant-associated environments is therefore essential for designing effective surveillance and stewardship strategies. Integrating agricultural systems into AMR monitoring frameworks may improve early detection of resistance emergence and support interventions that address transmission across environmental and public health sectors (FAO/OIE/WHO, 2020; Van Bruggen et al., 2019).

### 4.1 The one health framework: integrating plant pathology

The one health framework provides an integrated approach for addressing AMR by recognizing the interconnectedness of human, animal, plant, and environmental health systems. Within this framework, plant pathology contributes a critical perspective by addressing resistance selection and dissemination processes occurring in agricultural ecosystems (Miller et al., 2022; Van Bruggen et al., 2019). Plant disease management practices, including the use of antibiotics, fungicides, and other crop-protection agents, can influence microbial community composition and create selective pressures that favor resistant microorganisms. These changes may affect the abundance, persistence, and mobility of ARGs within plant-associated microbiomes, including the soil, rhizosphere, and phyllosphere. Such environments can serve as reservoirs of ARGs and facilitate their maintenance and horizontal transfer among environmental microorganisms, potentially contributing to broader resistance dissemination pathways (Zhu et al., 2013; Singer et al., 2016). Integrating plant pathology into AMR surveillance programs can enhance the detection of environmental resistance hotspots and improve our understanding of resistance dynamics across agricultural landscapes. Furthermore, promoting responsible antimicrobial use, integrated disease management, and ecosystem-based crop protection strategies can help mitigate resistance development while supporting sustainable agricultural production and environmental health (Food and Agriculture Organization of the United Nations, 2021, 2021; WHO, 2024).

## 5 India as a case study for AMR surveillance in agricultural systems

To provide a regional perspective, India’s AMR surveillance initiatives are highlighted as an example of progress and remaining challenges in agricultural systems under the one health framework. It has strengthened its AMR response through national sureveillance programs and multisectoral cocordination involving the Indian Council of Medical Research (ICMR), Indian Council of Agricultural Research (ICAR), and international partners thereby enhancing surveillance capacity and laboratory network (Indian Council of Medical Research, 2022; Indian Council of Agricultural Research, 2023; Food and Agriculture Organization of the United Nations, 2021).

Within agricultural systems, increasing attention has been directed toward monitoring antimicrobial residues, ARGs and environmental dissemination pathways associated with crop production and irrigation practices. Studies suggest that the use of untreated or contaminated wastewater may facilitate the movement of ARGs and resistant microorganisms into agricultural environments, with potential implications for food safety and ecosystem health (Fito and Van Hulle, 2021; Li et al., 2018; Marti et al., 2013). Despite these advances, AMR surveillance in plant production systems remains less developed than in the clinical and veterinary sectors. Similar challenges exist in many countries, highlighting the global need to strengthen crop-health surveillance, residue monitoring, and environmental AMR datasets to support integrated one health Surveillance.

## 6 Strengthening AMR surveillance and policy framework in agriculture

In India, the focus of monitoring and policy frameworks for AMR has traditionally been on human and animal health, with less emphasis placed on agriculture. The Government of India (2017) National Action Plan on AMR (NAP-AMR) for 2017–2027 emphasizes the importance of a one health approach that integrates human, animal, and environmental health. Initially, the plan concentrated on antimicrobial use in humans and livestock, but recent expert recommendations indicate that the crop sector should be explicitly included in order to address significant gaps in AMR monitoring and management with regard to plant diseases (Miller et al., 2022; Food and Agriculture Organization of the United Nations, 2021, 2021). The Indian Council of Agricultural Research (ICAR) has led efforts to address this surveillance gap by monitoring antimicrobial residues and resistant bacteria in soils and crops. These initiatives involve systematic sampling across different agro-ecological zones to detect residues of antibiotics such as streptomycin and oxytetracycline, and to characterize ARGs in bacteria associated with plants. Such measures require collaboration between the agricultural, environmental, veterinary and public health sectors in line with one health principles to combat AMR effectively.

## 7 Emerging mitigation and sustainable management strategies

**Figure 2:**
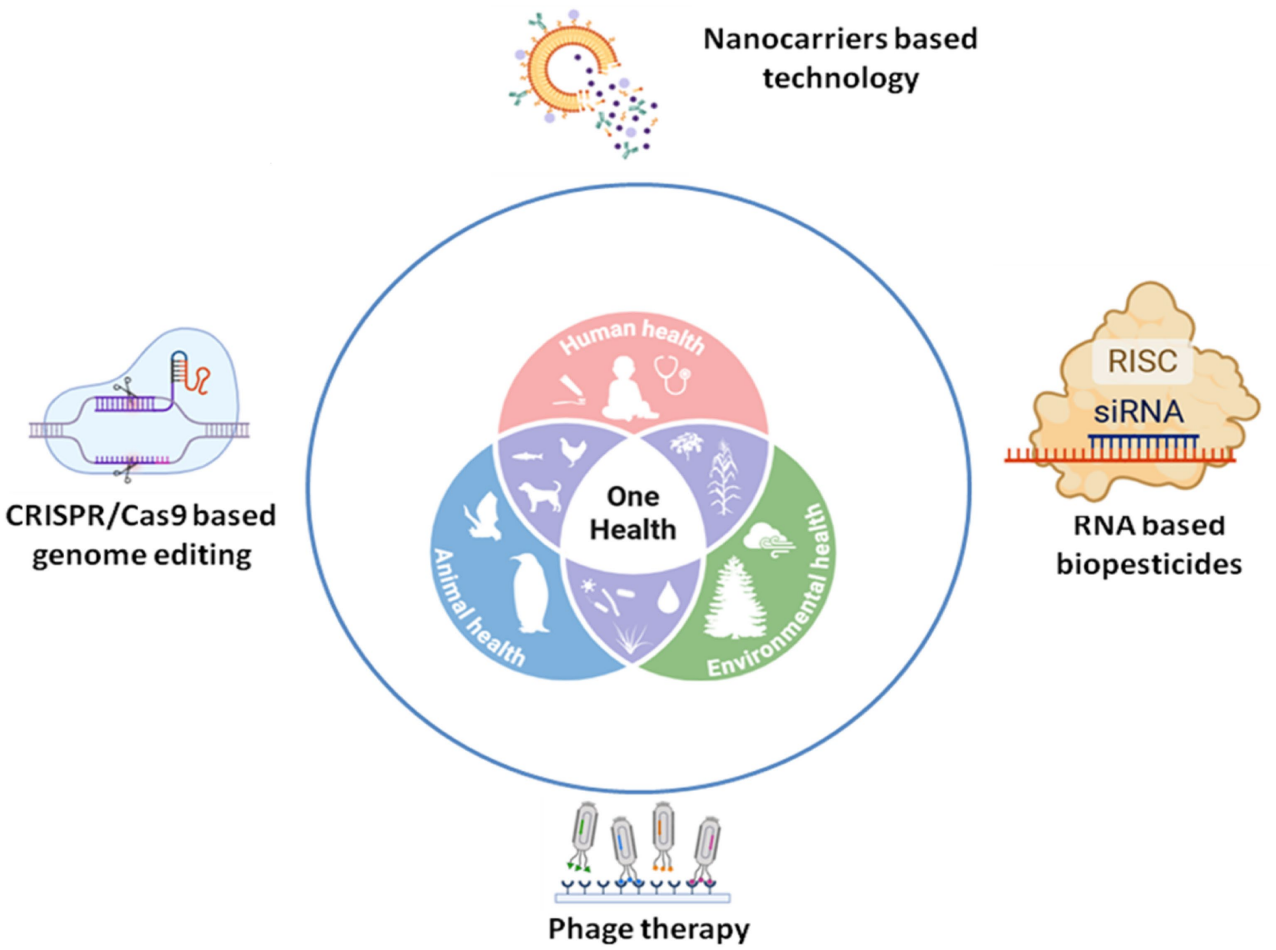
One Health framework for sustainable strategies to mitigate antimicrobial resistance in agriculture. The diagram illustrates the integration of human, animal, and environmental health within the One Health framework and highlights sustainable approaches to mitigate antimicrobial resistance, including CRISPR/Cas9 genome editing, RNA-based biopesticides, nanocarrier technology, and bacteriophage therapy.

Mitigating AMR in plant–pathogen interactions necessitates a paradigm shift away from relying solely on synthetic bactericides and fungicides toward integrated and sustainable disease management approaches. In recent times, various tools such as microbiome engineering, phage therapy, RNA-based biopesticides and nanotechnology-enabled disease management have provided sustainable, broad-spectrum alternatives that decrease the selection pressure for resistance while maintaining crop productivity (Table 2; Figure 2).

**Table 2:** Sustainable strategies for mitigating antimicrobial resistance in agriculture.

S. no	Strategy	Mode of action	Relevance for AMR mitigation in agriculture	One health implications	Reference
1.	Phage therapy	Bacteriophages infect and lyse plant pathogenic bacteria; phage cocktails increase host range and reduce resistance emergence.	Reduces antibiotic use in crops (e.g., fire blight, bacterial spots); targets pathogens with high specificity; low ecological disruption.	Limits ARG selection in plant environments; minimizes antibiotic residues entering soil, water, and food chains.	Svircev et al. (2018) and Grinter et al. (2016)
2.	RNA based biopesticides	dsRNA or siRNA silences essential genes in pathogens/insects; SIGS allows topical application; HIGS uses plant-expressed RNA molecules.	Offers pesticide-free disease control; prevents fungicide overuse; reduces evolution of fungicide resistance.	Biodegradable, highly specific, no residue risk; minimizes environmental AMR spread through selective targeting.	Huang et al. (2025) and Qiao et al. (2024)
3.	Microbiome engineering	Manipulation of beneficial rhizosphere/phyllosphere microbes; synthetic communities (SynComs); soil microbiome restoration	Enhances natural disease suppressiveness; reduces chemical inputs; outcompetes resistant pathogens.	Promotes soil and environmental health; reduces ARG enrichment and horizontal gene transfer in agroecosystems.	Carrión et al. (2019) and Vorholt et al. (2017)
4.	Nanotechnology	Green-synthesized Ag, Cu, ZnO, S nanoparticles; nano-encapsulation for controlled release; nano-enabled diagnostics	Low-dose, multi-target action reduces fungicide/bactericide use; delays resistance evolution; enhances efficacy of biocontrols.	Minimizes chemical residues; potential to lower environmental contamination; requires ecotoxicity assessment for safety.	Carrillo-Lopez et al. (2024) and Shafqat et al. (2023)
5.	CRISPR/Cas9 based genome editing	Editing susceptibility (S) genes; enhancing resistance genes; targeting pathogen genomes or ARGs; CRISPR diagnostics.	Provides durable, chemical-free resistance; reduces reliance on antibiotics and fungicides; targets resistance genes directly.	Lowers agricultural AMR burden; helps control pathogen spread; supports precision one health surveillance.	Bhalerao et al. (2023) and Bikard et al. (2014)

### 7.1 Microbiome engineering

The plant microbiome includes rhizosphere, phyllosphere and endosphere which are key players in nutrient acquisition, providing tolerance to abiotic stresses and natural disease suppression. A recent study in microbiome engineering provides a core strategy for sustainable plant protection and enhances the conventional plant disease management (Mueller and Sachs, 2015; Toju et al., 2018). Microbiome engineering comprises of selection and introduction of beneficial strains as bioinoculants, assemblage of SynComs, and transplantation of microbes through employment of various agronomic and cultural practices. SynComs are the microbial consortia which are involved in suppression of disease causing microbes in controlled conditions (Vorholt et al., 2017). These SynComs are mixed with various plant growth promoting rhizobacteria (PGPR) which includes *Bacillus, Pseudomonas* and *Streptomyces* and beneficial fungi include *Trichoderma* mainly and includes various antagonists that produce compounds like volatile organic compounds (VOCs), lytic enzymes, siderophores and biosurfacants that suppresses the growth of pathogens by competing for niche and resources thereby it secrets antimicrobial metabolites and induce systemic resistance in host plants (Carrión et al., 2019; Mendes et al., 2011). These SynComs targets various soil borne pathogens like *Ralstonia solanacearum*, *Fusarium* spp. and *Pythium* spp. showed reduction in the incidence of wilt and damping off through alterations in root exudates patterns and thereby it enhances the disease suppressive taxa. In recent times, in addition to rhizosphere SynComs now phyllosphere SynComs were being developed to control the foliar diseases through various approaches like pre-emption and by excluding competitive microbes in leaf habitats (Bai et al., 2015; Vorholt et al., 2017). Reduced tillage, reduced pesticide use, and the application of organic amendments and cover crops promote disease-suppressive soils by enhancing microbial diversity and beneficial microbial interactions. These practices suppress pathogen growth and reproduction while reducing the selection pressure that drives the emergence and dissemination of AMR (Hartmann et al., 2014).

Microbiome engineering represents a promising strategy for mitigating AMR by reducing dependence on synthetic bactericides and fungicides while promoting natural disease suppression through beneficial microbial interactions. However, its large-scale implementation faces several challenges. The effectiveness of introduced microbial strains or SynComs is often inconsistent under field conditions because their establishment, persistence, and colonization are influenced by indigenous microbial communities, plant genotype, soil characteristics, and climatic conditions. In addition, the development of stable formulations, including spore and encapsulation based technologies, remains essential for improving shelf life, field performance and large scale application. Regulatory approval of microbial consortia, production costs, scalability, quality control, and the lack of standardized application protocols further limit their widespread adoption. Recent advances in metagenomics, gnotobiotic systems, microbial community modelling, and formulation technologies are expected to improve the design, stability, and field performance of microbiome-based interventions, thereby supporting their integration into sustainable and resistance-resilient plant disease management strategies (Toju et al., 2018; Mueller and Sachs, 2015; Carrión et al., 2019).

### 7.2 Phage therapy

Bacteriophages (phages) are viruses that specifically infect bacterial hosts and have emerged as a potential alternative or complementary strategy to conventional antimicrobials for managing bacterial plant diseases (Balogh et al., 2010; Jones et al., 2012). Their interest in crop protection stems from their high host specificity and self-replicating nature, which may enable suppression of target pathogens while reducing unintended effects on non-target microbial communities. Phage therapy has been explored for controlling bacterial diseases including fire blight of apple, bacterial speck, bacterial canker, and bacterial wilt. The antimicrobial activity of lytic phages involves adsorption onto susceptible bacterial cells followed by genome injection, replication using host cellular machinery, and eventual bacterial cell lysis with release of progeny phages that can infect neighboring pathogen populations (Svircev et al., 2018). Several phage-based formulations have been developed against agriculturally important pathogens, particularly *Xanthomonas* spp. and *E. amylovora*, with applications focused primarily on foliar disease management (Jones et al., 2012).

Recent developments have emphasized the use of phage cocktails, where multiple phages are combined to broaden host range and reduce the probability of rapid resistance evolution. Such approaches have shown potential in systems involving *E. amylovora*, *X. campestris* pv. *campestris*, and *P. syringae* pathosystems (Balogh et al., 2010; Svircev et al., 2018; Pinheiro et al., 2019). Additional formulation strategies including encapsulation in protective polymers and integration with complementary approaches such as copper-based compounds, botanical products, and beneficial microorganisms have been investigated to improve phage persistence under field conditions and enhance disease suppression (Iriarte et al., 2007; Balogh et al., 2010; Pinheiro et al., 2019; Svircev et al., 2018).

Although phage therapy offers advantages including target specificity and potentially reduced selection pressure on non-target microbiota, its practical implementation remains constrained by several biological and operational limitations. Environmental factors such as ultraviolet radiation, temperature fluctuations, rainfall, and desiccation can substantially reduce phage survival and efficacy under field conditions. Furthermore, narrow host range, variability in pathogen populations, emergence of phage-resistant bacterial strains, and inconsistency between greenhouse and field performance remain major challenges (Grinter et al., 2016; Ghequire and De Mot, 2014; Koskella and Brockhurst, 2014; Javeedvali et al., 2024). Regulatory approval pathways, formulation standardization, and scalability for large-scale agricultural deployment also remain insufficiently developed.

### 7.3 RNA based biopesticides

RNA-based biopesticides have emerged as a promising experimental and early stage alternative approach for plant disease management by exploiting the natural RNA interference (RNAi) pathway to suppress genes associated with pathogen virulence, survival, or host colonization (Rani et al., 2024; Huang et al., 2025; He et al., 2022; Zhao et al., 2025). These technologies involve the exogenous delivery of double-stranded RNA (dsRNA) molecules that trigger sequence-specific degradation of target messenger RNA, thereby reducing pathogen fitness while minimizing broad-spectrum effects associated with conventional antimicrobial inputs. The major implementation strategies include Host-Induced Gene Silencing (HIGS) and Spray-Induced Gene Silencing (SIGS). HIGS relies on transgenic expression of RNA molecules within the host plant, whereas SIGS involves external application of dsRNA formulations onto plant surfaces to induce gene silencing in target organisms. Among these approaches, SIGS has received increasing research attention because it does not require modification of the crop genome and externally applied RNA molecules generally exhibit limited environmental persistence, potentially simplifying regulatory acceptance in some jurisdictions (Huang et al., 2025). Experimental and proof of concept studies have demonstrated the potential of dsRNA-mediated suppression of virulence-associated pathways, including genes involved in effector production, cell wall formation, signal transduction, and sterol biosynthesis. Disease reduction and decreased mycotoxin accumulation have been reported in pathosystems involving *Fusarium graminearum*, *Botrytis cinerea*, powdery mildew, and rust pathogens (He et al., 2022; Sundararajan et al., 2025). These findings suggest that RNA-based approaches have the potential to reduce reliance on conventional antimicrobial inputs in crop protection.

Despite these promising outcomes, several limitations currently restrict large-scale agricultural deployment. Environmental instability remains a major challenge because dsRNA molecules are susceptible to degradation by ultraviolet radiation, nucleases, rainfall, and variable field conditions, resulting in inconsistent efficacy and limited persistence after application (Qiao et al., 2024). Efficient uptake by plant tissues and target pathogens also remains incompletely understood and varies across species and environmental contexts. To improve delivery efficiency, nanocarrier platforms including layered double hydroxides, chitosan nanoparticles, liposomes, and polymer-based carriers have been investigated to enhance dsRNA protection, foliar retention, and cellular uptake (Qiao et al., 2024). However, these formulation technologies introduce additional considerations regarding production cost, scalability, environmental fate, and regulatory evaluation.

At present, only a limited number of RNA-based biopesticides have advanced toward commercialization. Their broader adoption in agricultural will depend on improving formulation stability and delivery efficiency, reducing production costs, demonstrating consistent performance under diverse field conditions obtaining regulatory approval, and establishing long-term environmental safety (Zhao et al., 2025; Huang et al., 2025).

### 7.4 Nanotechnology based disease management

Nanotechnology has emerged as a potential tool for plant disease management through applications in pathogen detection, targeted delivery of active compounds, and improved formulation efficiency. Nanomaterials possess properties such as high surface area, tunable physicochemical characteristics, and controlled release behavior, which may improve the precision and effectiveness of disease management strategies while potentially reducing dependence on conventional chemical inputs (Carrillo-Lopez et al., 2024). Recent advances include the development of green nanotechnology, in which plant extracts and microorganisms are used as reducing and stabilizing agents during synthesis of metal and metal oxide nanoparticles, with the objective of lowering environmental impact relative to conventional production methods (Wasule et al., 2024). Several nanoparticle formulations have demonstrated antimicrobial activity under experimental conditions. For example, silver nanoparticles have shown inhibitory effects against bacterial pathogens including *Xanthomonas*, *Pseudomonas*, and *Ralstonia*, as well as fungal pathogens such as *Botrytis*, *Alternaria*, and powdery mildew-associated fungi, primarily through mechanisms involving membrane disruption and oxidative stress induction (Shafqat et al., 2023). Similarly, copper- and copper oxide-based nanoparticles have been investigated as lower-dose alternatives to conventional copper formulations, while zinc oxide nanoparticles have shown potential activity against soil-borne pathogens including *Fusarium* and *Phytophthora* species (Vijayalakshmi et al., 2025).

Beyond direct antimicrobial activity, nanotechnology has also been explored as a delivery platform. Nano-encapsulation approaches may improve the performance of fungicides, botanical compounds, and emerging biopesticides by enhancing solubility, protecting active ingredients from degradation, and enabling more controlled release profiles that reduce application frequency (Irshad et al., 2024; Sandhya et al., 2025). In addition, nanobiosensors, quantum dots, and nano-enabled diagnostic platforms have been proposed to improve early pathogen detection and support timely disease intervention, potentially reducing prophylactic pesticide applications (Carrillo-Lopez et al., 2024; Sandhya et al., 2025).

Despite these opportunities, the long-term contribution of nanotechnology to AMR mitigation remains uncertain. Although reduced chemical application rates may decrease selection pressure in some contexts, current evidence is insufficient to conclude that nanomaterials directly delay resistance evolution. Important concerns remain regarding nanoparticle persistence, environmental accumulation, toxicity to non-target organisms, impacts on beneficial microbiota, and unintended alterations to soil and plant-associated microbial communities (Carrillo-Lopez et al., 2024). Furthermore, nanoparticle performance observed under controlled conditions may not consistently translate to field-scale agricultural systems. In addition, large-scale adoption is constrained by production costs, formulation stability, regulatory approval, and the need for comprehensive risk assessments to ensure environmental and human safety.

### 7.5 CRISPR based solutions

The recent CRISPR based solutions provide promising opportunities for sustainable plant disease management and represent a potential alternative strategy to mitigate AMR in agriculture. CRISPR technology enables precise genetic modifications in plants, pathogens and associated microbiomes, thereby reducing dependence on antibiotics and fungicides while lowering selection pressure that contributes to AMR development in agricultural ecosystem. Within one health framework, CRISPR based innovation target the genetic determinants of disease susceptibility, pathogen virulence and microbial community dynamics, offering long-term and environmentally sustainable disease management solutions.

Experimental studies have demonstrated that CRISPR-mediated editing of plant susceptibility (S) genes can enhance resistance against several pathogens by disrupting host factors required for pathogen infection, thereby reducing reliance on chemical disease management. Targeted knockout or modification of these genes can confer durable and broad-spectrum resistance while minimizing chemical inputs. Proof-of-concept studies have shown that CRISPR–Cas systems can selectively target ARGs bearing plasmids and disrupt pathogen virulence-associated genes, indicating their potential for reducing resistant microbial populations while preserving beneficial microorganisms (Bikard et al., 2014). Future applications may include CRISPR-enabled gene drives and genome engineering approaches to manipulate pathogen populations. However, these concepts remain largely theoretical in plant pathology and require extensive experimental validation before practical implementation, particularly for pathogens such as *Botrytis cinerea* and *Fusarium* spp. (Bhalerao et al., 2023). CRISPR-mediated engineering of biocontrol microorganisms, including *Bacillus* and *Pseudomonas*, has also shown potential to enhance production of antifungal metabolites and improve biological disease suppression (Li et al., 2025).

Furthermore, CRISPR-guided editing strategies can facilitate targeted removal or disruption of ARGs, suppressing undesirable microbial pathways and potentially reducing resistance selection pressure (Rubin et al., 2020). Recent advances in CRISPR-based diagnostics have enabled rapid detection of single-nucleotide mutations associated with fungicide resistance in pathogens such as *Zymoseptoria tritici* and *Botrytis cinerea*, supporting timely disease surveillance and management decisions (Kaminski et al., 2021).

Despite its considerable potential, several challenges currently limit the widespread application of CRISPR-based technologies in agricultural disease management. Efficient delivery of CRISPR components into plants, pathogens, and associated microbiomes remains technically challenging, particularly under field conditions. Concerns regarding off-target genome modifications, editing efficiency, and the long-term stability of edited traits require further investigation. In addition, regulatory frameworks governing genome-edited crops and microorganisms vary considerably among countries, which may delay commercialization and field deployment. Public acceptance, production costs, biosafety considerations, and the need for extensive field validation across diverse agroecological conditions also remain important barriers. Continued advances in genome editing technologies, delivery systems, and regulatory harmonization are expected to improve the safe and effective implementation of CRISPR-based approaches for sustainable disease management and AMR mitigation.

Overall, CRISPR-based technologies represent a promising approach for sustainable plant disease management and have the potential to reduce reliance on antimicrobial inputs in agricultural systems. Experimental studies and proof-of-concept investigations suggest that these technologies may contribute to mitigating AMR by targeting disease susceptibility, pathogen virulence, and ARGs. However, further field-scale validation is required to determine their long-term effectiveness, environmental impact, and practical contribution to limiting AMR dissemination in agricultural ecosystems. These advances are consistent with the broader goals of the one health framework to promote sustainable disease management and strengthen AMR stewardship.

## 8 Challenges and future directions

### 8.1 Surevillance and diagnostic gaps

AMR in plant associated microbes includes complex scientific, ecological and regulatory challenges which highlight the need for coordinated one health interventions. Although AMR has been extensively studied in human and veterinary sectors, its emergence and dissemination in crop production systems remain less well understood. These unresolved issues hinder the development of integrated, sustainable strategies that will mitigate the AMR risks that arise in the field of plant pathology. The key challenges arises due to limited surveillance and data deficit, lack of routine diagnostics, limited molecular detection techniques for screening esistant alleles, absence of monitoring system in soil, water and phyllosphere microbiomes.

### 8.2 Regulatory and policy limitations

Lack of regulatory and policy framework leads to accessibility and availability of antibiotics and copper fungicides which enables the usage and thereby it exerts the selection pressure. After application, the long term residual effect is the major concern which results in accumulation in soil and irrigation systems which alters the soil microbial community, enrichment of ARGs and enables the HGT among beneficial and pathogenic microbes which harness the ecological link between crops based AMR and human or animal interface which can be recognized intensively. Various transmission routes include irrigation water, dust, aerosols and compost but these routes are unexplored. Understanding these interfaces in the near future will predict downstream impacts on one health. Plant pathogens including fungi, bacteria showed high mutation rates, recombinations and frequent gene transfer horizontally and the repeated chemical usage showed increased resistance emergence mainly against specific site of action fungicides like strobilurins, DMIs.

### 8.3 Sustainable alternatives

Future systems must include resistome profiling in soils, rhizosphere, phyllosphere and monitoring of antimicrobial residual level in agricultural ecosystem, surveillance for resistance alleles in the pathogen and should include data on plant derived AMR into national one health AMR platforms. Adaptation of alternatives that reduce the dependency on chemicals so that it exerts minimal selection pressure through phage mediated biocontrol, microbiome engineering to maintain suppressive soils, RNA based biopesticides for targeting specific pathogens through RNAi mechanism, nanotechnology based formulations and CRISPR based genome editing must be integrated to ensure long term durability and biocompatibility. One should evaluate the potential for resistance development before approving the chemicals; promote low risk alternatives and their integration into pest management strategies so that chemical residues or footprints can be minimized through policy support and farmers training. Strengthening environmental and food chain biosecurity by assessing AMR transfer via fresh produce, post harvest environment and supply chain, employment of biosafety measures to restrict the movement of resistant plant pathogens.

### 8.4 Future research priorities

In the future studies on mechanism behind shaping of ARG persistence in soil and plant microbiome, their interactions with various pesticides, fertilizers and their impact on pathogen evolution and AMR dynamics will help in identifying the AMR hotspots for better management strategies. To improve the generation and dissemination of AMR data at global level, interdisciplinary collaboration among plant pathologists, microbiologists, ecologists, veterinarians and health professionals is essential to translate scientific knowledge into effective implementation at national and global scales.

## 9 Conclusion

AMR in plant-associated microbial communities represents an emerging challenge with implications for agricultural productivity, environmental sustainability, and the broader one health framework. This review highlights that antimicrobial use in crop production, together with ecological and genetic mechanisms of resistance dissemination, contributes to the persistence and spread of antimicrobial resistance within agricultural ecosystems. Strengthening surveillance of plant-associated antimicrobial resistance, improving stewardship of antimicrobial compounds, and integrating sustainable disease management approaches, including biological and emerging molecular technologies, will be critical for reducing selection pressure and preserving their long-term effectiveness. Advancing interdisciplinary research, evidence based policies, and cross-sector collaboration will be essential to incorporate plant health into global one health strategies and support resilient and sustainable agricultural systems.
